# Magneto-Electricity in Disease

**Published:** 1844-03

**Authors:** 


// THE WESTERN JOURNAL.

New Series.—Vol. I.—No. III.

LOUISVILLE, MARCH 1, 1844.

### MAGNETO-ELECTRICITY IN DISEASE.

The readers of this Journal will remember a reference, made in a former number, to the success of Dr. Walkly, of Tuscaloosa, Alabama, in the treatment of various complaints by *magneto-electricity.* We received from Dr. Walkly, a short time since, a pamphlet, of nearly sixty pages, giving a history of fifty-six cases in which he applied this agent with decided benefit. His experience in the use of the remedy has now extended through upwards of two years, in which time he has tried it in cases of rheumatism, paralysis, leucorrhœa, dysmenorrhœa, amaurosis, epilepsy, hysteria, intermittent fever, neuralgia, and amenorrhœa. Of the latter complaint Dr. W. says, his journal contains a record of *forty* cases, "all of which have been successfully treated by means of electricity, and in a much shorter time than under the usually prescribed course of medicine." One or two applications, just before the expected period, generally brought on the catamenia in recent cases of dysmenorrhœa. He has not seen mischief result from the application of electricity, where the suppression depended upon pregnancy, in two cases of which he resorted to it without suspecting that pregnancy was the cause. His practice is to pass "a rapid succession of shocks through the uterus, until pain is felt in the lumbar region, and this is induced in from five to ten minutes by placing the negative electrode directly above the pubis, and the positive to the spinal nerves of the lower lumbar vertebræ." Another mode adopted by many, which has some advantages, is to place one conductor in a warm bath in which the feet of the patient are immersed, and the other to the back of the neck.

Dr. Walkly's success in the employment of this remedy seems to have been decided. In seven cases of amaurosis, out of eleven, he states that the relief from it was complete, one was benefitted for a time, and only three remained unimproved. He reports cases of *angina pectoris,* without, however, insisting upon the diagnosis, which he cured by electricity. Asthma, also, it seems, has yielded to its influence, and that too where the disease had been of long standing. "I have," he says' "treated eleven cases of *chorea,* all of which were cured, with a single exception. No other remedy was used, except occasional aperients." Out of seventeen cases of *deafness* in which he resorted to it, eight are said to have been entirely relieved.

This, certainly, was extraordinary success, and if anything approaching it can be attained generally, magneto-electricity must soon come to be regarded as among the most efficient of our curative measures. Making all due allowance for the ardor of one who, probably, is riding his hobby hard enough, we are inclined to give great credit to Dr. Walkly for the perseverance with which he has applied his favorite remedy to so many forms of disease. Nor can we close this notice without expressing the hope, that others will engage in the same interesting field of experiment.

Excellent magneto-electrical machines are made in this city by Mr. Jacob Walter & Son. Y.

